# Rio1 mediates ATP-dependent final maturation of 40S ribosomal subunits

**DOI:** 10.1093/nar/gku878

**Published:** 2014-10-07

**Authors:** Tomasz W. Turowski, Simon Lebaron, Elodie Zhang, Lauri Peil, Tatiana Dudnakova, Elisabeth Petfalski, Sander Granneman, Juri Rappsilber, David Tollervey

**Affiliations:** Wellcome Trust Centre for Cell Biology, University of Edinburgh, Michael Swann Building, Kings Buildings, Mayfield Road, Edinburgh EH9 3JR, UK

## Abstract

During the last step in 40S ribosome subunit biogenesis, the PIN-domain endonuclease Nob1 cleaves the 20S pre-rRNA at site D, to form the mature 18S rRNAs. Here we report that cleavage occurs in particles that have largely been stripped of previously characterized pre-40S components, but retain the endonuclease Nob1, its binding partner Pno1 (Dim2) and the atypical ATPase Rio1. Within the Rio1-associated pre-40S particles, *in vitro* pre-rRNA cleavage was strongly stimulated by ATP and required nucleotide binding by Rio1. *In vivo* binding sites for Rio1, Pno1 and Nob1 were mapped by UV cross-linking in actively growing cells. Nob1 and Pno1 bind overlapping regions within the internal transcribed spacer 1, and both bind directly over cleavage site D. Binding sites for Rio1 were within the core of the 18S rRNA, overlapping tRNA interaction sites and distinct from the related kinase Rio2. Site D cleavage occurs within pre-40S-60S complexes and Rio1-associated particles efficiently assemble into these complexes, whereas Pno1 appeared to be depleted relative to Nob1. We speculate that Rio1-mediated dissociation of Pno1 from cleavage site D is the trigger for final 18S rRNA maturation.

## INTRODUCTION

The yeast pre-ribosome processing and assembly pathway takes place in both the nucleus and cytoplasm. Three nuclear pre-rRNA cleavages, at sites A0, A1 and A2 generate the 20S pre-rRNA within large complexes termed 90S pre-ribosomes or SSU processomes. The 20S pre-rRNA is exported to the cytoplasm in pre-40S particles, within which it undergoes final maturation. Over 200 proteins and some 75 small nucleolar RNAs (snoRNAs) function as cofactors during the complex pathway of ribosomal subunit maturation. During this process a large number of structural changes and RNA processing events occur, but mechanistic understanding of these steps has been hampered by a lack of tractable *in vitro* systems for biochemical analyses.

One exception is the very late step in 40S subunit maturation in which the 3′ end of the 20S pre-rRNA is cleaved at site D, by the PIN-domain endonuclease Nob1, to generate the mature 18S rRNA (1,2). As first shown for human Smg6 (3), all tested eukaryotic PIN-domain nucleases show a dependency on Mn^2+^ for RNA cleavage *in vitro*. The levels of Mn^2+^ required (∼2 mM) are non-physiological, and the dependence of this class of enzyme on divalent cations *in vivo* remains unclear (2–7). However, this provided a useful tool, since the pre-ribosomes could be purified in the presence of Mg^2+^, and then activated for pre-rRNA cleavage by addition of Mn^2+^ (8).

Previous analyses revealed that *in vitro* cleavage at site D within pre-40S particles is stimulated by addition of either adenosine triphosphate (ATP) or GTP to the reaction (8). The relevant GTPase was identified as the translation initiation factor eIF5b (Fun12 in yeast) (8), but the putative ATPase protein was not identified. Three proteins that associate with late pre-40S particles appeared to be likely candidates; the DEAH-box RNA helicase Prp43, and the atypical protein kinases Rio1 and Rio2. Prp43 has multiple roles in ribosome synthesis, including the stimulation of site D cleavage (2). Rio1/Rrp10 and Rio2 are related to each other and well conserved in evolution, but show low sequence homology to other protein kinase families (9,10). Both proteins are required for site D cleavage in yeast and human cells *in vivo* (11–14). Structural and functional analyses of Rio2 revealed that an autocatalytic activity, and/or subsequent hydrolysis of an aspartylphosphate residue generated in the active site, is required for dissociation of Rio2 from pre-ribosomes (15). Rio2 binds in the core of the 18S rRNA region (16) and can bind to Nob1 and the late-binding 40S synthesis factor partner of Nob1 Pno1/Dim2 *in vitro* (17). Less is known about the interactions made by Rio1 but its kinase activity is required for late pre-40S maturation in human cells, suggesting that its function may be mechanistically similar to Rio2.

In contrast to the early assembly events, the cytoplasmic steps in the maturation of ribosomal subunits largely involve the disassembly of the many non-ribosomal proteins that mediated RNA processing and ribosomal protein integration. This suggested that only the last protein(s) to leave the pre-ribosomes should remain associated with cleavage-competent particles. Here we show that, among the late-binding pre-40S components, only this property is strongly shown only by Rio1 and Pno1, in addition to the Nob1 endonuclease. We conclude that the major, cleavage-competent pre-40S complexes have lost most of the characterized ribosome-synthesis factors, and retain only Rio1, Pno1 and the Nob1, in addition to the ribosomal proteins.

## MATERIALS AND METHODS

### Strains and media

All yeast analyses were performed in strains derived from (BY4741, *MAT*a; *his3Δ1*; *leu2Δ0*; *met15Δ0*; *ura3Δ0*). Strains used are listed in Supplementary Table S1.

### Pre-40S purification and characterization

Purification of pre-40S particles, fractionation by gel filtration and *in vitro* cleavage were performed as described (8). Primer extension assays were performed as described (18). To assess protein phosphorylation upon *in vitro* cleavage conditions ATP was replaced with radiolabeled [γ-^32^P] ATP at 64 nM.

### Protein digestion and LC-MS/MS analysis

Trichloroacetic acid-precipitated proteins were dissolved in 50 μl 8 M urea, proteins were reduced with 1 mM DTT for 1 h at room temperature (RT) and alkylated with 5 mM iodoacetamide for 1.5 h at RT. Initial digestion was performed with 1 μg LysC (Wako, 129–02541) for 4–5 h at RT, urea concentration was then diluted to <2 M with 180 μl 50 mM ammonium bicarbonate and final digestion was performed with 1 μg trypsin for another 12 h at RT. The reaction was stopped by the addition of 24 μ of 10% TFA, further diluted with 200 μl 0.1% TFA and peptides were desalted on manually packed C18-StageTips (3× 16-gauge Empore C18 plugs per tip). Eluted peptides were dried down, reconstituted in 0.1% TFA and analyzed by LC-MS/MS on a UltiMate 3000 RSLCnano LC coupled to a Q Exactive mass-spectrometer platform (Thermo Scientific). Peptides were loaded on a spray emitter (75 μm ID, 360 um OD, 30 cm in length, New Objective) packed with 3 μm Reprosil-Pur C18-AQ beads (Dr. Maisch GmbH) and separated over a 80-min gradient of 2–40% B (A, 0.1% formic acid in water; B, 0.1% formic acid in 80% acetonitrile), followed by a 10 min gradient of 40–95% B and a 5 min wash at 95% B. The Q-Exactive was operated in a data-dependent top 10 acquisition mode, MS1 scans were recorded over m/z range of 350–1400, with a resolution of 70 000 and AGC target of 1e6 ions. After each full scan, up to 10 MS2 scans were recorded, with a resolution of 17 500, AGC target of 5e4, maximum injection time of 60 ms and NCE of 25. Fragmented ions were dynamically excluded for 20 s, all scans were recorded in profile mode and Q Exactive was operated with Exactive 2.2 software.

### Proteomic data analysis

Raw data files (.raw files) were converted to peak lists using the MSConvert routine from ProteoWizard 3.0 package, a database search was performed with Mascot 2.4.1 (Matrixscience). In brief, peak lists were searched against a *Saccharomyces cerevisiae* sequence database downloaded from UniProtKB on 20.04.2013, with Trypsin/P as protease, Carbamidomethylation (C) as fixed and Oxidation (M) as variable modification. The decoy search option was used to estimate FDR (false discovery rate). Precursor ion mass tolerance was set at 6 ppm, fragment ion tolerance was set at 0.02 Da. Identified protein lists were filtered to 1% FDR level by Mascot. The resulting .dat data files were used to build spectral library in Skyline 2.5 (University of Washington), which was then used to perform MS1 Full-Scan Filtering (19). Individual sample fractions were treated as single replicates and only peptides found across all samples were used to calculate total protein intensity. Proteins were quantified using the spectral counting quantification-exponentially modified protein abundance index (emPAI) (see 20).

### Crosslinking and analysis of Illumina sequence data

CRAC experiments using Rio1-HTP, Pno1-HTP and Nob1-HTP were performed on cultures grown in SD medium with 2% glucose, lacking Trp to OD_600_ 0.5. Cells were cross-linked in culture media (21) and processed as previously described (16,22). Illumina sequencing data were aligned to the yeast genome using Novoalign (http://www.novocraft.com). Cluster generation used Cluster 3.0 and Java Treeview. Downstream analyses were performed using the pyCRAC tool suite (23) and the PyMOL Molecular Graphics System, Version 1.5.0.4 Schrödinger, LLC.

## RESULTS

### Cleavage-competent pre-ribosomes are preferentially associated with Rio1

A key feature of the late steps in 40S ribosomal subunit maturation is the dissociation of the ribosome synthesis factors that are required for earlier assembly processes. This suggested that factors that are associated with intermediate processes would not be present in the final stages of maturation and so would not co-precipitate with late, cytoplasmic particles. We hypothesized that cleavage-competent particles should include only the Nob1 nuclease and the very last factor(s) to bind or be released.

The pre-40S particles that contain the 20S pre-rRNA and accumulate in the cytoplasm have been extensively characterized by biochemical analyses and electron microscopy 3D reconstruction (8,16,24–26,27). Pre-40S complexes were purified by precipitation of tagged forms of each of the known late pre-40S associated ribosome synthesis factors; Enp1, Ltv1, Tsr1, Dim1, Pno1, Nob1 and the ATPases, Prp43, Rio1 and Rio2. All of the tagged proteins were shown to support growth, showing they are largely or completely functional. In each case the pre-40S particles were purified in buffer containing 5 mM MgCl_2_ and immobilized on immunoglobulin G columns using the Protein A moiety of the affinity tag. With exception of tagged Nob1 strain, endogenous Nob1 is present in the purified pre-40S particles. In particles that have reached a cleavage-competent state, Nob1 can be activated to cleave the endogenous 20S pre-rRNA by exchanging the purification buffer, which contains Mg^2+^, for buffer containing 5 mM MnCl_2_, with the addition of 1 mM ATP or GTP.

**Figure 1.**
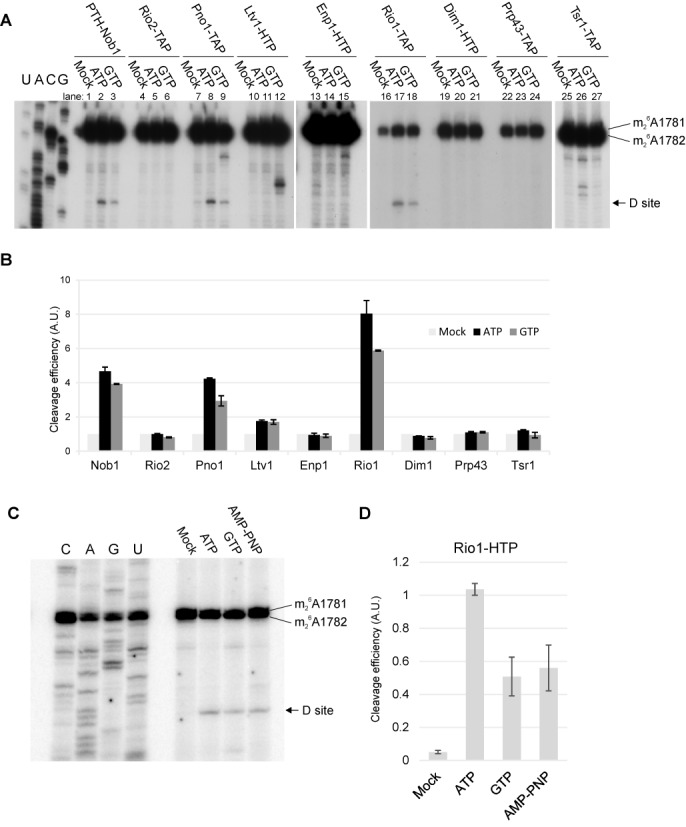
Pre-ribosomes associated with Rio1 or Pno1 undergo efficient cleavage. (A) Primer extension analyses of site D cleavage in pre-40S particles purified by association with the indicated bait proteins. The gels show the relative cleavage efficiencies in the absence of added nucleotides (mock) or with addition on 1 mM ATP or GTP. (B) Quantitation of cleavage efficiency. Cleavage analyses as in panel A were performed in three biological replicates. Cleavage efficiency was determined by the ratio of the primer extension stops site D and at 18S+1781/1782, and is expressed relative to the mock-treated sample incubated in the absence of added ATP or GTP. Error bars show ±1 SD. (C) Comparison of cleavage in Rio1-associated pre-ribosomes, in the presence of ATP, GTP or non-hydrolysable AMP-PNP each at 1 mM. (D) Quantitation of cleavage efficiency. Cleavage efficiency was calculated as in B except that ratio is expressed relative to the ATP-treated sample and error bars show standard error.

Following incubation, RNA was extracted and analyzed by primer extension through cleavage site D (Figure 1A). In Figure 1B, the degree of cleavage stimulation by nucleotide addition is quantified with cleavage efficiency assessed relative to the primer extension stop at the methylation sites A1781/1782 in 18S rRNA. Specific cleavage at site D was not observed in pre-ribosomes associated with Rio2 or Prp43, in the presence or absence of ATP or GTP, and was also not seen for Enp1, Dim1 or Tsr1. In the case of Ltv1, the graph indicates some degree of cleavage, however, inspection of the gels indicates that this apparent stimulation reflects a very low level of basal cleavage in the mock-treated sample without added NTP. As previously observed, Nob1 copurifies with cleavage competent pre-ribosomes (Figure 1A, lanes 1–3), as does the Nob1-binding protein Pno1 (Figure 1A, lanes 7–9). However, cleavage was clearly most efficient in pre-ribosomes copurified with the Rio1 kinase (Figure 1A, lanes 16–18). We interpret this as showing that Nob1 and Pno1 are largely recovered in association with earlier, cleavage-non-competent pre-40S particles in addition to late, cleavage-competent pre-ribosomes. Rio1 binds later and is therefore preferentially associated with the cleavage competent complexes.

We conclude that Rio1 is the only known ATPase that remains associated with cleavage-competent pre-40S particles. Notably, pre-ribosomes associated with the related Rio2 kinase showed no cleavage activity, strongly indicating that Rio2 is released from the pre-ribosomes before Rio1.

### Pre-rRNA cleavage in Rio1-associated particles is stimulated by ATP

To assess whether pre-rRNA cleavage in the Rio1-associated pre-40S particles requires ATP hydrolysis, we compared stimulation by ATP with the non-hydrolysable analog AMP-PNP and GTP (Figure 1C and D). Addition of each of the nucleotides stimulated site D cleavage, but stimulation by ATP was clearly greater than with AMP-PNP, indicating that ATP hydrolysis is important for cleavage activity. This difference was seen in 10 independent experiments. Addition of AMP-PNP or GTP conferred similar cleavage stimulation, suggesting that this might be the result of NTP binding, rather than hydrolysis, either to Rio1 or an additional protein.

The catalytic activity of Rio1 was shown to regulate its pre-40S association (28). To assess whether nucleotide binding or ATP hydrolysis by Rio1 is responsible for the observed ATPase dependence, two point mutations were generated. Based on the yeast Rio1 structure (28) K125R lies in the predicted ATP biding site, altering a critical residue that is highly conserved in the protein kinase family and plays a key role in orienting the α and β phosphate moieties of ATP (reviewed in (29)). D244A alters the catalytic aspartic acid, and blocks autophosphorylation and casein phosphorylation activities *in vitro* (10).

**Figure 2.**
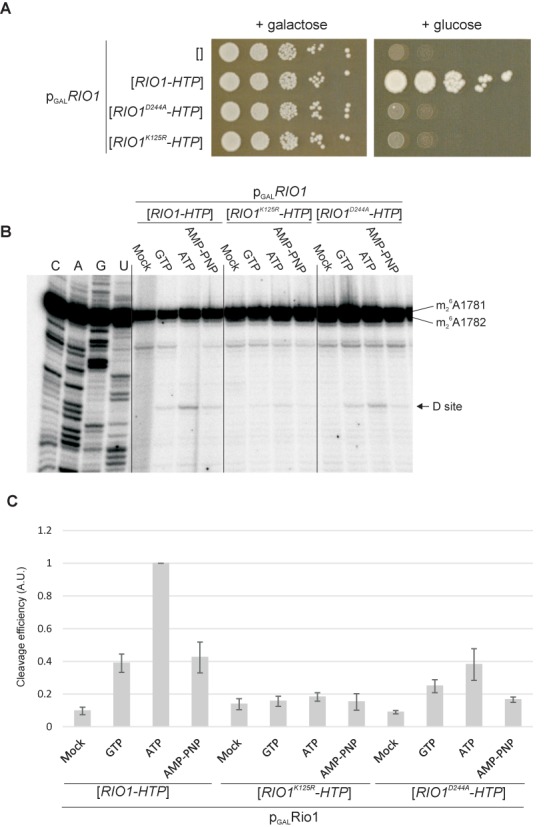
The ATP binding ability of Rio1 is required for growth and site D cleavage. (A) Growth of *P_GAL_::RIO1* strain on permissive galactose medium or repressive glucose medium, without plasmid (empty brackets) or complemented by plasmids expressing functional Rio1-HTP or Rio1 with the D244A or K125R mutations. (B) Comparison of cleavage in pre-ribosomes associated with Rio1, Rio1^K125R^ and Rio1^D244A^, in the presence of ATP, GTP or non-hydrolysable AMP-PNP each at 1 mM. (C) Quantitation of cleavage efficiencies. Cleavage efficiency was expressed relative to the ATP-treated sample for functional Rio1. Error bars show standard error.

To test the effects of these mutations, we generated a *P*_*GAL*_*::RIO1* strain, in which chromosomal *RIO1* gene is expressed on galactose medium but repressed on glucose medium. This strain was transformed with plasmids expressing Rio1 or Rio1_K125R_ or Rio1_D244A_ carrying C-terminal His6-TEV-Protein A (HTP) tags. Growth tests (Figure 2A) showed that expression of Rio1-HTP supported growth of the *P_GAL_::RIO1* strain on glucose medium, whereas neither the K125R nor D244A mutant proteins complement the growth of the Rio1-depleted strain. Pre-ribosomes were purified from the wt and mutant Rio1 strains and tested for *in vitro* cleavage activity (Figure 2B). Interestingly, the catalytic mutant form of Rio1 (D244A) only partially reduced D cleavage efficiency *in vitro*, whereas the K125R mutation in the ATP binding pocket almost completely abolished cleavage, showing that nucleotide binding by Rio1 is required for cleavage.

Rio2 was shown to be an atypical protein kinase, which undergoes autophosphorylation that is linked to its release from pre-ribosomal particles (15). However, this activity is not manifested in pre-ribosomal particles copurified with Rio2, apparently due to occlusion of the active site (15). It seemed possible that autophosphorylation by Rio1 might be coupled the acquisition of cleavage competence. To test for autophosphorylation, purified pre-40S particles associated with Rio1 or Rio2 were incubated under cleavage conditions, in the presence of [γ^32^P] ATP (Supplementary Figure S1). No clear protein phosphorylation was observed in Rio1-associated pre-40S particles. The Rio2-associated pre-ribosomes showed robust protein phosphorylation, which was previously shown to be due to the presence of the casein kinase II homolog Hrr25 (15). The lack of this activity in Rio1-associated particles is consistent with the loss of most 40S synthesis factors from the later Rio1-associated particles. We conclude that Rio1 does not exhibit clear autophosphorylation activity in pre-40S particles.

### Rio1-associated pre-ribosomes form 80S particles

Previous analyses established that site D cleavage is associated with formation of 80S-like complexes, in which the pre-40S particles bind mature 60S ribosomal subunits (8,25,30). The preferential association of cleavage-competent pre-ribosomes with Rio1-TAP suggested that Rio1 should also be preferentially associated with pre-80S particles.

**Figure 3.**
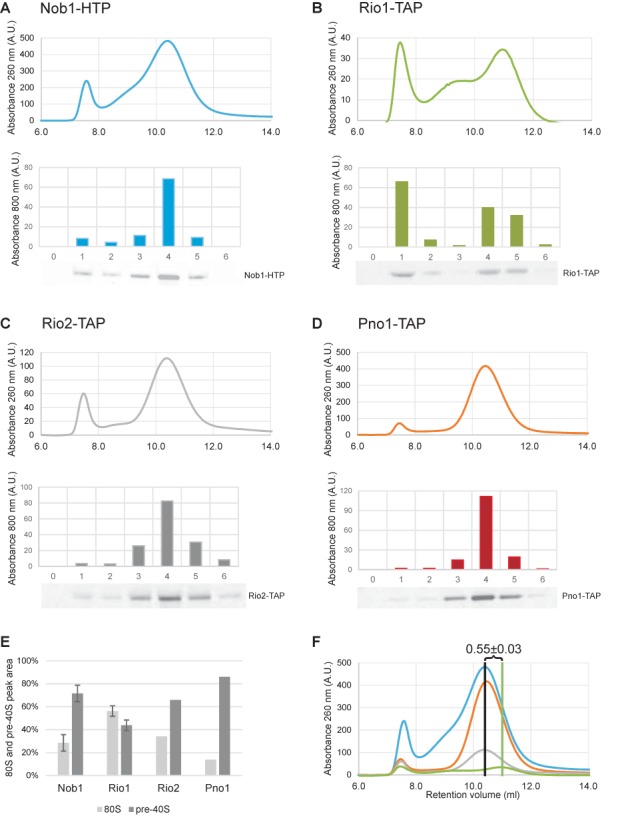
Rio1-associated pre-ribosomes form 80S complexes. Pre-40S particles purified using Nob1-HTP (blue), Rio1-TAP (green), Rio2-TAP (gray) and Pno1-TAP (orange) were analyzed by size exclusion chromatography (top of panels A–D). Comparison to a calibration curve (not shown) revealed that the two major peaks correspond to 80S particles and 40S particles as previously reported (8). The distribution of tagged proteins in chosen fractions was assessed by western blot and quantified using a Licor Odyssey system (bottom of panels A–D). 80S and pre-40S peak areas were quantified and plotted as percent of its sum (E). Results of different experiments were superimposed presenting difference in retention volume of pre-40S peaks.

The size distribution of particles associated with Rio1, Rio2, Pno1 and Nob1 was assessed by gel filtration (Figure 3). The Superose 6 column used has a separation range of up to 5 MDa and complexes of ∼80S are therefore expected to elute in fraction 1. As previously observed (8), Nob1-associated particles were distributed between 40S and 80S fractions of the column eluate (Figure 3A). However, a substantially higher fraction of Rio1-associated particles eluted in the 80S fractions (Figure 3B). In contrast, lower recovery of 80S particles was seen for Rio2 and Pno1 (Figure 3C and D). The data are quantified in Figure 3E. This was supported by primer extension analyses of the 18S and 25S rRNA, and 20S pre-rRNA, present in the fractions are presented in Supplementary Figure S2. The chromatograms are superimposed in Figure 3F, showing that the major ‘40S’ peak associated with Rio1 is of lower apparent molecular weight than those associated with the other proteins. This is consistent with the hypothesis that the Rio1-associated complex is smaller than the major forms of the other pre-ribosomal complexes and adopts a more compact conformation as recently reported (31). The amount of material recovered with Rio1 was notably less than for Nob1 and Pno1, suggesting that only a small fraction of the pre-ribosomes that are associated with Nob1-Pno1 are also associated with Rio1. This is also the case for Rio2, although the yield of Rio2-associated pre-ribosomes was greater than for Rio1.

It was previously shown that the ‘80S’ peak in the Nob1-associated material largely represents Nob1-bound pre-40S particles joined together with 60S ribosomal subunits (8,25). Western analyses showing the distribution of Nob1 confirmed that this is also the case in the experiments reported here (Figure 3A). However, it is likely that some level of earlier 90S pre-ribosomes is also present, since Nob1 associates with these particles prior to nuclear export. The distribution of the other bait proteins across the elution profile was also assessed by western blotting (Figure 3B–D). The distribution of Rio2 was similar to Nob1, whereas Pno1 was different, with a substantially weaker signal in the 80S fractions. For Rio1 a stronger peak was observed in the 80S fraction than for other factors tested, confirming that these particles are formed with Rio1-bound pre-40S particles. This further indicates that pre-40S particles associated with Rio1 are more frequently competent for 80S complex formation than are particles associated with Nob1, Pno1 or Rio2. Moreover, the lower recovery of Pno1 relative to Nob1 in the 80S fractions, suggested that Pno1 may dissociate prior to release of Nob1.

### Mass-spectrometry of proteins associated with pre-ribosomal complexes

**Figure 4.**
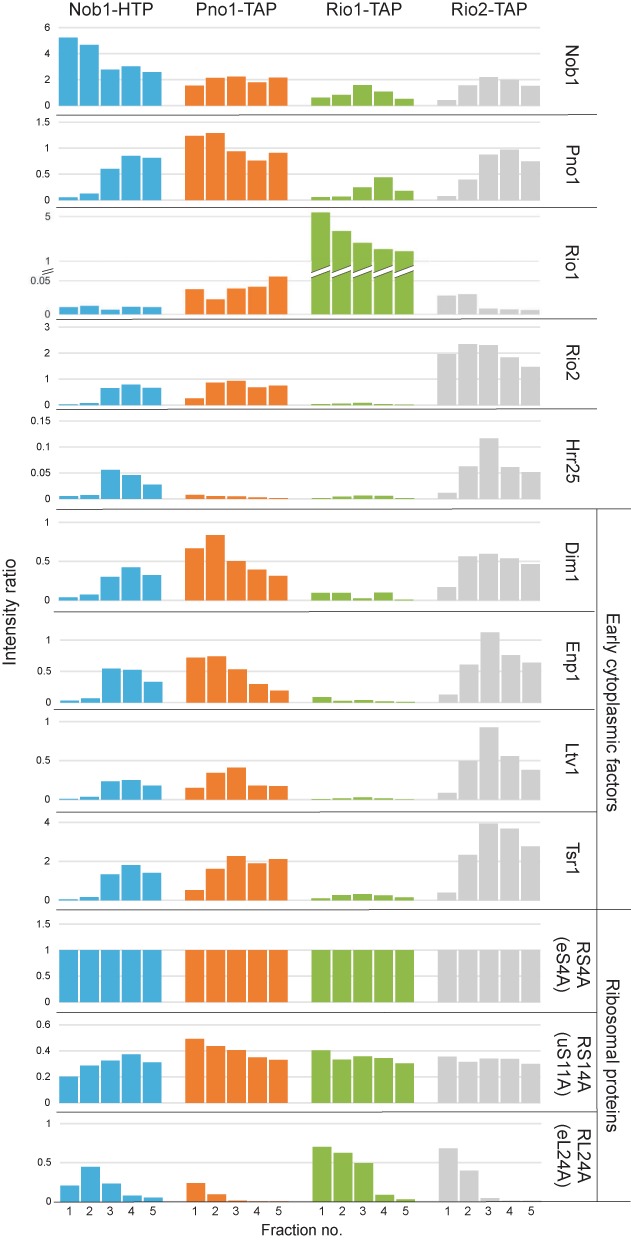
Mass-spectrometry of proteins associated with pre-ribosomal complexes. Fractions 1–5, previously tested by western blotting (Figure 3), were analyzed by mass spectrometry. Plots show ratios of summed-up peptide intensities for Nob1, Pno1, Rio1 and Rio2, Dim1, Enp1, Ltv1 and Tsr1, expressed relative to 40S subunit ribosomal protein Rps4A.

To further characterize proteins associated with these pre-ribosomal complexes, elution fractions 1–5 from size exclusion chromatography of the Nob1, Pno1, Rio2 and Rio1 samples were subjected to trypsin digestion and mass-spectrometry. Supplementary Table S2 lists the identified proteins, while data normalized to the abundance of ribosomal proteins are shown in Figure 4. Names that correspond to the recently proposed unified nomenclature for ribosomal proteins (32) are given on in parentheses. The 40S peak (fractions 4 and 5) from the Nob1, Pno1 and Rio2 gel filtration columns contained the major pre-40S associated proteins, including Enp1, Tsr1, Dim1 and Ltv1. These fractions therefore largely contain the previously characterized, major pre-40S particles (16,24). In contrast, in the Rio1-associated particles contained only low levels of 40S synthesis factors, with the exception of Rio1, Nob1 and Pno1. In the Pno1 and Nob1 precipitates Rio1 is substoichiometric, as expected if it binds to only the very last particles that contain Pno1 and Nob1. Rio1 appears superstoichiometic in the Rio1 precipitate, and this might indicate that Rio1 is transiently retained in 80S complexes following release of both Nob1 and Pno1 as recently suggested (28).

The 80S complexes associated with Nob1, Pno1, Rio1 and Rio2 also contained the common snoRNP proteins, which are readily detected because multiple snoRNPs are present in each pre-ribosome. A pronounced shoulder of higher mass material was associated with the 40S peak in the Rio1 precipitate. We considered that this might represent pre-40S particles that were associated with the GTPase Fun12/eIF5b. However, this region apparently lacked significant amounts of Fun12, but contained 60S ribosomal proteins (Supplementary Table S2).

We conclude that Rio1 is preferentially associated with pre-40S complexes that contain only Rio1, Nob1 and Pno1, in addition to the small subunit ribosomal proteins.

### Identification of the *in vivo* binding sites for Rio1, Pno1 and Nob1

To better understand their interactions with pre-40S particles the binding sites were mapped by ultraviolet (UV) cross-linking and analysis of cDNAs (CRAC) in actively growing cells. For this, Rio1 and Pno1 were expressed as C-terminal fusions with the tripartite His6-TEV-Protein A from the chromosomal loci, under control of the endogenous promoters. The binding site for Nob1 in pre-40S particles was previously identified in cells that were intact but non-growing, as they had been harvested in phosphate buffered saline and held on ice during irradiation (16). Techniques for *in vivo* cross-linking in actively growing cells have subsequently been developed (21), allowing transient, dynamic interactions with maturing pre-ribosomes to be better recovered.

**Figure 5.**
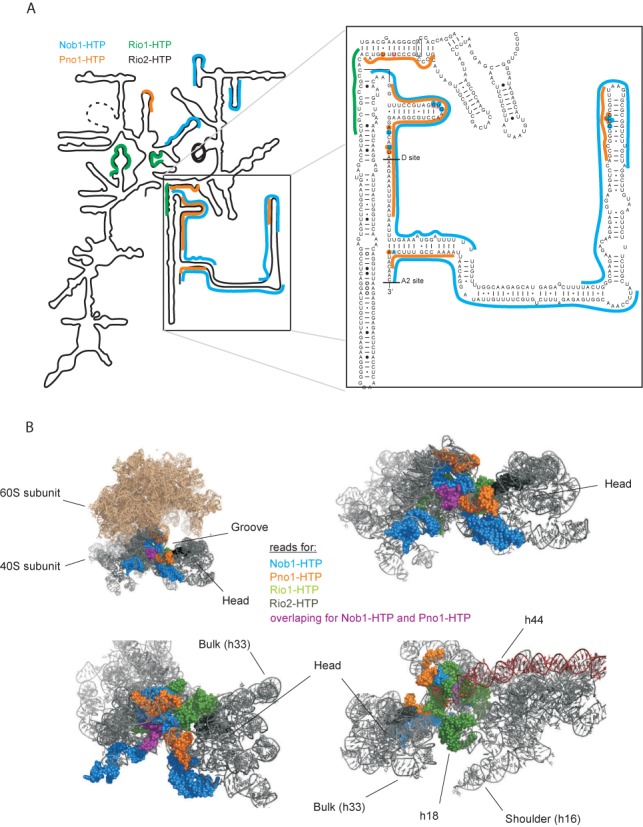
Pre-40S rRNA binding sites identified for Nob1, Pno1 and Rio1. (A) Mapped reads for Nob1-HTP (blue), Pno1-HTP (orange) and Rio1-HTP (green) *in vivo* on a simplified 20S pre-rRNA secondary structure. Inset: Sequence and secondary structure of the 3′ region of the 18S rRNA and 5′ region of ITS1. Nucleotides identified as direct protein contact sites are highlighted. (B) 18S rRNA derived from a crystal structure of the yeast 80S ribosome (PDB ID 3U5B and 3U5D). Sequences identified in CRAC analyses described here are indicated in space-filling mode and colored to indicate binding proteins: Nob1-HTP (blue), Pno1-HTP (orange) and Rio1-HTP (green). Reads common to Nob1-HTP and Pno-HTP, in the 3′ region of 18S rRNA are marked with purple. The crystal structure lacks the ITS1 regions that are bound by Nob1-HTP and Pno1-HTP.

Binding sites for Rio1 were identified in the core of the 18S rRNA structure (Figure 5A shows an outline of the 18S rRNA secondary structure; the full 18S sequence is shown in Supplementary Figure S3A). Notably, the Rio1 binding sites were distinct from the previously identified binding site for Rio2, indicating that these related proteins do not successively occupy the same location on the pre-rRNA. Nob1 was cross-linked to H40 in the 18S rRNA, as previously reported (Figure 5A and B) (16). However, cross-linking in actively growing cells also identified further interaction sites in 18S, in the 5′ domain of ITS1 and over cleavage site D (Figure 5A and B). We conclude that cross-linking of actively growing cells captures more transient, but functionally important, interactions. Pno1 (Partner of Nob1) was initially identified as a Nob1 binding protein (33) and showed a pattern of bindin g in ITS1 that was strikingly similar to that of Nob1. However, the binding sites for Pno1 and Nob1 were distinct within the 18S rRNA region (Figure 5B).

**Figure 6.**
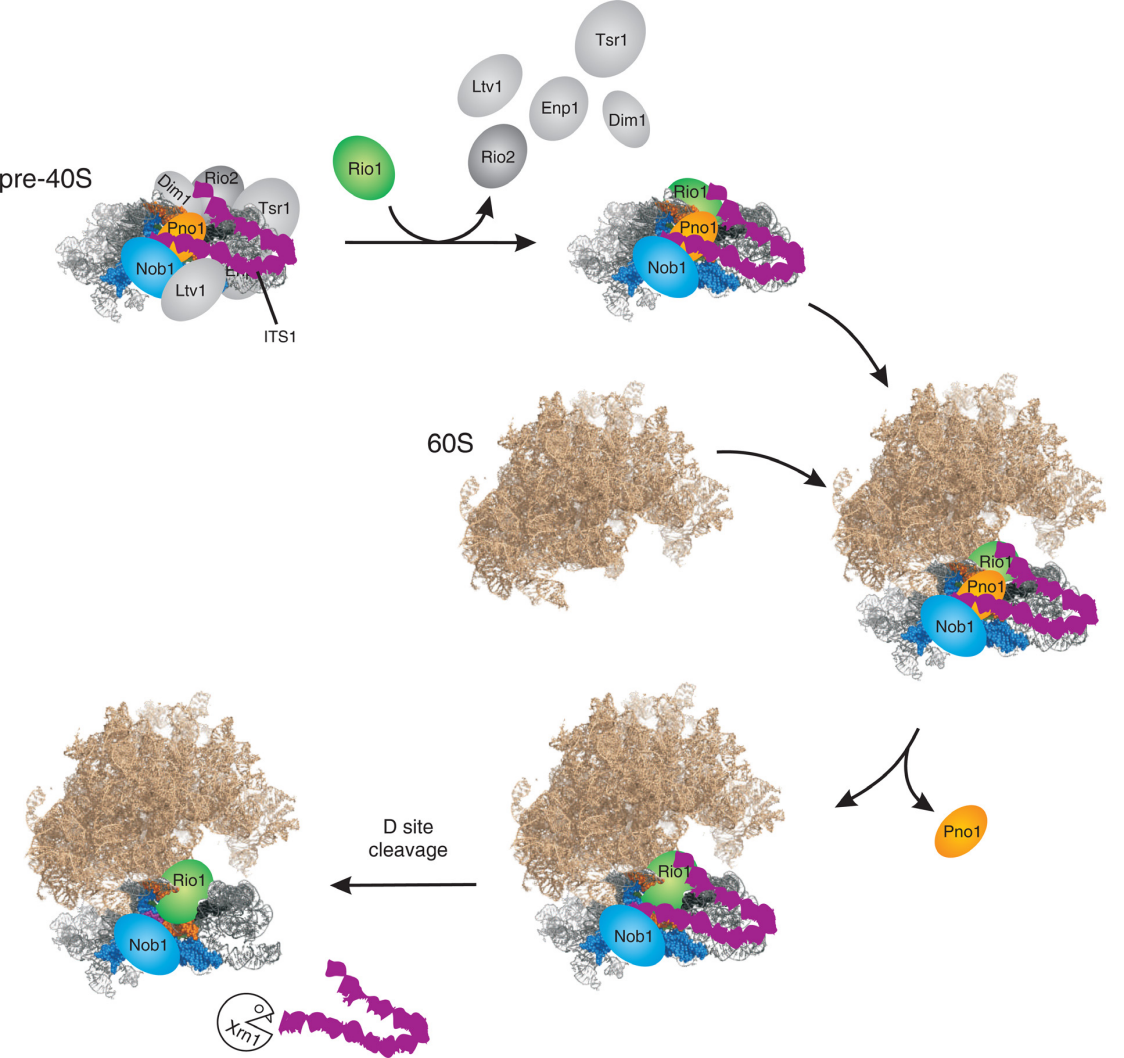
Model for late pre-40S processing. The starting point is the previously characterized, major cytoplasmic pre-40S particle. As the particle matures, individual factors pre-40S assembly factors dissociate; Ltv1, Enp1, Tsr1, Dim1 and Rio2. However, Nob1 and Pno1 are retained and Rio1 binds to the particle. Following association with the mature 80S subunit, Pno1 is lost from site D, which is then cleaved by Nob1. As indicated schematically, the long stem structure of the 5′ region of ITS1 is predicted to fold back and to be held in close proximity to the body of the late pre-40S particle. Following D site cleavage, ITS1 is rapidly released and is degraded by the cytoplasmic 5′ exonuclease Xrn1.

The 5′ domain of ITS1, from site D to site A2, is predicted to form a very long stem structure (Figures 5A and 6). Large regions of the 5′ domain of ITS1 could be recovered associated with Pno1 and Nob1 (Figure 5A) indicating that this region is substantially protected against RNase digestion by protein binding and/or pre-rRNA folding. This indicates that ITS1 does not simply extend out from the pre-40S particles. Within these regions, specific protein contact sites were identified by point mutations in the cDNA sequences (22,34), and these locations are indicated by highlighted nucleotides in Figure 5A.

Conserved sequence elements required for the function of ITS1 are located adjacent to sites D and A2, and at the terminus of the stem structure (35). These functionally important locations are in strikingly good agreement with the Nob1-Pno1 binding sites, and no other proteins have been reported to bind these sequences in the pre-rRNA. This strongly suggests that their conserved role is the binding of Nob1 and Pno1. Notably, Nob1 and Pno1 each bind to nucleotides located both in the base and at the extremity of this extended stem. This makes it very likely that it adopts a fold-back structure, perhaps folding at the prominent bulge that is drawn as a hinge in Figure 6.

## DISCUSSION

Ribosome subunit synthesis proceeds through a series of high complexity RNA-protein particles, most of which are difficult to analyze in structural detail. Following nuclear export, pre-40S particles that accumulate in the cytoplasm contain the 20S pre-rRNA and a limited number of ribosome synthesis factors, Enp1, Ltv1, Tsr1, Dim1, Pno1/Dim2, Rio2 and Nob1, with less stable association of Prp43. The comparative stability and relative simplicity of these pre-40S particles compared to earlier, nuclear pre-ribosomes has made them popular targets for biochemical and structural analyses (16,24,27). Previous reports have envisaged that structural rearrangements within these particles provide the trigger for site D cleavage at the 3′ end of the 18S rRNA. Here, we report that pre-rRNA cleavage does not occur in these well-characterized particles, which are the precursors to simpler complexes apparently containing only the atypical protein kinase Rio1, the KH RNA binding domain protein Pno1 and the PIN domain endonuclease Nob1, in addition to the small subunit ribosomal proteins. These, largely disassembled, pre-40S particles join with 60S subunits prior to the acquisition of cleavage competence, which probably involves removal of Pno1 from cleavage site D (see Figure 6).

The efficiency of pre-rRNA cleavage seen in pre-ribosomes copurified with Rio1 was substantially greater than with any other ribosome synthesis factors tested, including the endonuclease Nob1. At first sight this might seem unexpected, but Nob1 binds nuclear pre-ribosomes prior to export to the cytoplasm, so only a small fraction of Nob1-associated pre-ribosomes can be cleavage-competent. This also explains why pre-40S particles co-purified with Nob1 and Pno1 are associated with number of early maturating factors (Figure 4). In contrast, our data indicate that Rio1 binds late pre-40S particles shortly before cleavage. In consequence, a larger fraction of the associated pre-ribosomes are expected to be in a cleavage-competent conformation. It was previously reported that Pno1 interacts with Nob1 and with Rio2 in an *in vitro* binding assay (26). Since the data clearly indicate that Rio2 is released prior to the acquisition of cleavage-competence, we speculate that a Pno1/Nob1/Rio2 complex exists in pre-40S particles immediately following export to the cytoplasm. This may be replaced by a Pno1/Nob1/Rio1 complex prior to cleavage of the 20S pre-rRNA. The presence of Pno1, in earlier, cleavage-incompetent pre-40S complexes together with Rio2 would correlate well with the reduced processing efficiency of Pno1-associated particles relative to Rio1.

Structural and functional analyses of Rio2 showed that ATP is used to generate a phosphoaspartate intermediate at Asp_257_ in the active site. Subsequent hydrolysis of the phosphoaspartate potentially triggers release of Rio2, and perhaps other ribosome synthesis factors. However, the active site of Rio2 is unavailable for ATP addition in the intact pre-40S particles (15). Similarly, we observed that, while ATP binding by Rio1 is apparently required for pre-rRNA cleavage in Rio1-associated pre-ribosomes, the addition of ^32^P-labeled ATP does not result in detectable radiolabeling of Rio1 or other pre-ribosome components.

Archaeal Pno1 is predicted to bind the 3′ end of the 16S rRNA (36), and this is also the case for the yeast protein. Indeed, all of the binding sites for Pno1 and Nob1 within internal transcribed spacer 1 (ITS1) were almost fully overlapped. These proteins are reported to form a complex, but it is unlikely that the overlapping RNA interaction sites simply reflect binding of the complex. The complex will be dissociated during the CRAC protocol, since proteins are purified in highly denaturing buffer containing 6M Guanidine HCl, and UV is a ‘zero-length’ cross-linker, so the protein and RNA have to be in direct contact for cross-linking to occur. The very extensive overlap between the Pno1 and Nob1 binding sites makes it most unlikely that these interactions could occur simultaneously. We therefore speculate that some form of RNA hand-over takes place. The obvious possibility is that Pno1 initially binds to ITS1 and site D but, in a late structural remodeling step, is replaced by Nob1 immediately prior to RNA cleavage. This would be consistent with the low recovery of Pno1 in the 80S complexes in which cleavage is believed to occur (Figures 3 and 4) (8,25).

A striking feature of most previous analyses of the RNA binding sites for pre-40S ribosome synthesis factors is the predominance of targets within the conserved core of the mature rRNAs. This was initially unexpected, because it had been widely assumed that eukaryotic rRNA expansion elements and transcribed spacer regions would provide specific protein-binding sites. Pno1 and Nob1 are therefore more unusual in having major binding sites within ITS1.

Previous analyses of the final steps in 40S ribosome subunit maturation have focused on the stimulation of 80S complex formation and cleavage by the GTPase activity of the translation factor eIF5b/Fun12 (8,25,30). However, Fun12 is not essential for growth or 40S subunit synthesis, strongly indicating that an alternative maturation pathway exists. Moreover, *in vitro* pre-rRNA cleavage could be stimulated by ATP, in addition to GTP (8). The data presented here show that the alternative pathway involves ATP binding by Rio1 and also promotes cleavage in 80S-like particles. Site D cleavage is more efficient when ATP hydrolysis occurs but ATP binding appears to be sufficient to support cleavage *in vitro* (Figure 2). It seems likely that the catalytic activity of Rio1 is connected with the release of mature ribosomal subunits following site D cleavage (28). The existence of alternative pathways that are at least partially redundant presumably enhances the overall efficiency and robustness of 40S subunit maturation. The interplay and relative contributions of the two pathways to ribosome synthesis *in vivo* remain to be determined.

## SUPPLEMENTARY DATA

Supplementary Data are available at NAR Online.

SUPPLEMENTARY DATA
