# Supplementary material for: Rio1 mediates ATP-dependent final maturation of 40S ribosomal subunits
Source: Nucleic Acids Res. 2014 Oct 7;42(19):12189–99. doi: 10.1093/nar/gku878 (PMC4231747; doi:10.1093/nar/gku878)
Supplement: SUPPLEMENTARY DATA [file supp_42_19_12189__index.html]

Rio1 mediates ATP-dependent final maturation of 40S ribosomal subunits — SUPPLEMENTARY DATA 

# Rio1 mediates ATP-dependent final maturation of 40S ribosomal subunits

## SUPPLEMENTARY DATA

**Files in this Data Supplement:**

- SUPPLEMENTARY DATA
