# Supplementary material for: Rio1 mediates ATP-dependent final maturation of 40S ribosomal subunits
Source: Nucleic Acids Res. 2014 Oct 7;42(19):12189–99. doi: 10.1093/nar/gku878 (PMC4231747; doi:10.1093/nar/gku878)
Supplement: SUPPLEMENTARY DATA [file supp_gku878_nar-01553-r-2014-File008.pdf]

SUPPLEMENTAL INFORMATION

Supplementary Table and Figure Legends

Supplementary Table S1: Yeast strains use in these analyses

Supplementary Table S2: Proteomic analyses of gel filtration column fractions

The table lists proteomic hits identified from column fractions 1-5 from samples coprecipitated with Rio1, Rio2, Pno1 and Nob1. Proteins in the table are filtered down to 1% FDR level and sorted by values obtained from spectral counting and quantification of the exponentially modified protein abundance index (emPAI) (see (Arike and Peil, 2014)). This provides approximate, label-free quantification of relative protein abundances. Data from these tables were used to prepare the graphs shown in Figure 5.

Supplementary Figure S1: Kinase reaction with [γ^{32}P] labeled ATP.

Comparison of phosphorylation of biogenesis factors by endogenous kinases activities associated with pre-40S particles isolated with Rio1-HTP, Rio2-HTP, Nob1-HTP and Dim2-HTP. Pre-rRNA cleavage in the isolated pre-40S particles was stimulated by addition of 5mM Mn^{2+} ions and [γ^{32}P] ATP. Proteins were separated by SDS-PAGE, transferred and followed by autoradiography of membranes. The prominent band arising form Hrr25 phosphorylation activity {Ferreira-Cerca, 2012 #3563} is indicated.

Supplementary Figure S2: Primer extension analysis of rRNA in gel filtration fractions.

A: Outline of the primer extension scheme used. To detect 20S pre-rRNA, the primer was located with the ITS1 region, adjacent to a long tract without cytosine residues. The reaction mixture contained dATP, dCTP and dTTP, together with ddGTP. Under these conditions, primer PE001 gives a defined product for 20S pre-rRNA.

B: Gel filtration plots with fractions analyzed in C indicated.

C: Primer extension analyses of the 5` ends of 18S and 25S rRNA and 20S of pre-rRNA.

Supplementary Figure S3: Pre-40S rRNA binding sites identified for Nob1, Pno1 and Rio1

A: Mapped reads for Rio1-HTP (green) and Rio2-HTP (black) {Granneman, 2010 #2706}, on the 20S pre-rRNA secondary structure.

B: Mapped reads for Nob1-HTP (blue) and Pno1-HTP (orange) *in vivo* on the 20S pre-rRNA secondary structure

Fig. S1

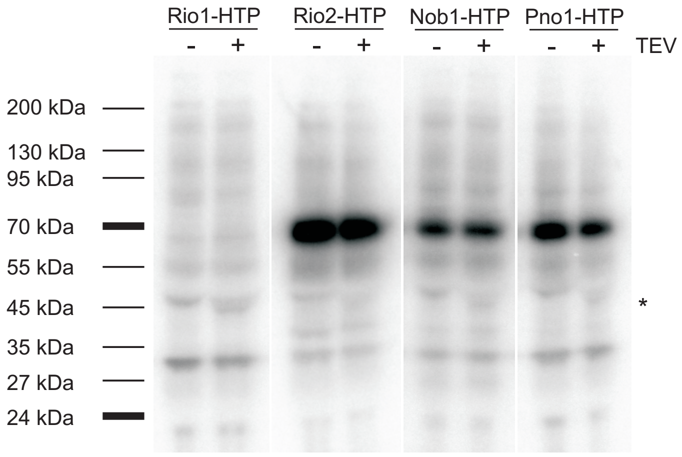
Rio1-HTP
Rio2-HTP
Nob1-HTP
Pno1-HTP
- + - + - + - + TEV
200 kDa
130 kDa
95 kDa
70 kDa
55 kDa
45 kDa
35 kDa
27 kDa
24 kDa
*

Fig. S2

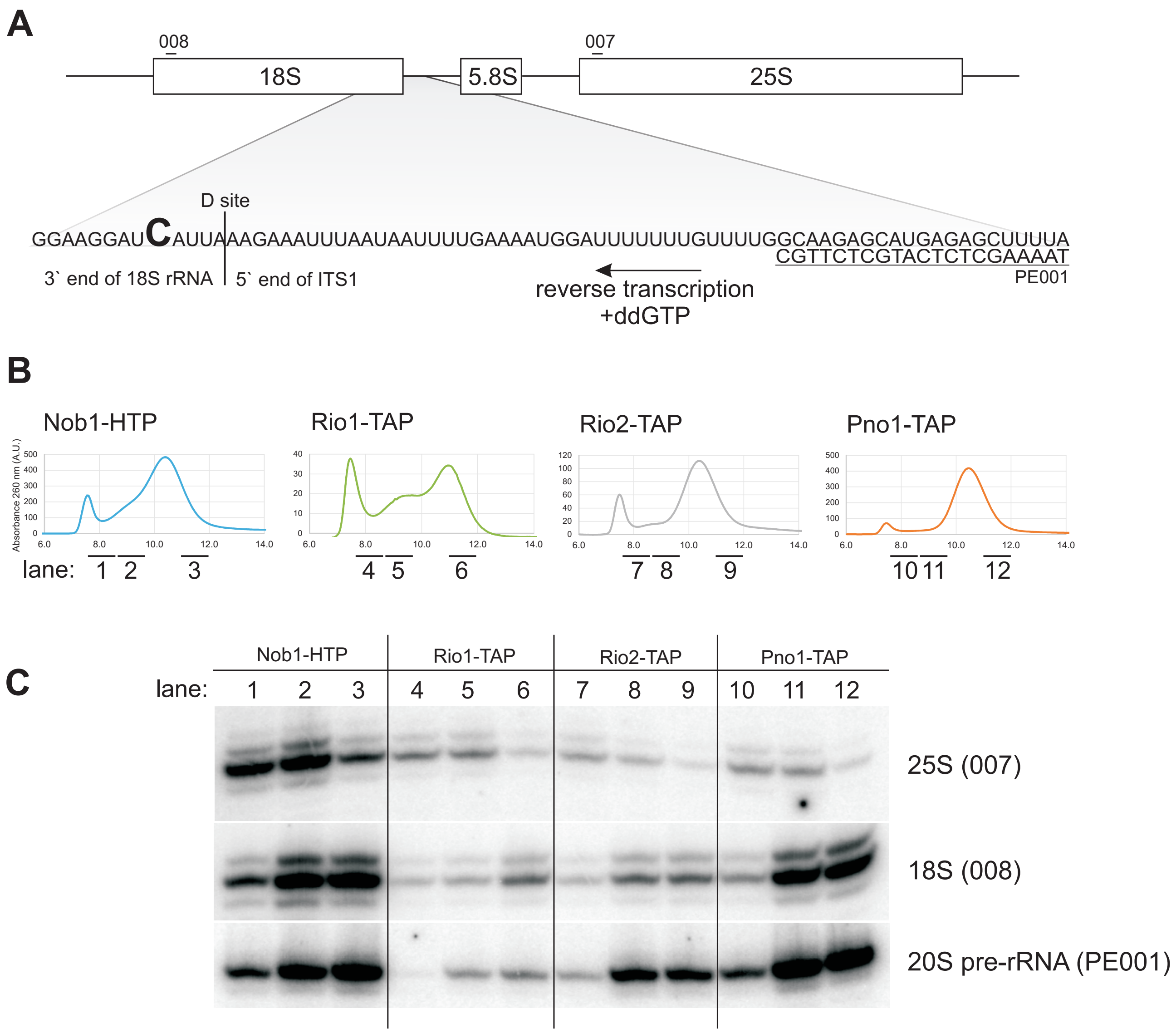
A
008
18S
5.8S
007
25S
D site
GGAAGGAUCAUUAAAGAAAUUUAAUAAUUUUGAAAAUGGAUUUUUUUGUUUUGGCAAGAGCAUGAGAGCUUUUA
CGTTCTCGTACTCTCGAAAAT
3` end of 18S rRNA
5` end of ITS1
reverse transcription
+ddGTP
PE001
B
Nob1-HTP
Rio1-TAP
Rio2-TAP
Pno1-TAP
Absorbance 260 nm (A.U.)
lane: 1 2 3 4 5 6 7 8 9 10 11 12
C
Nob1-HTP
Rio1-TAP
Rio2-TAP
Pno1-TAP
lane: 1 2 3 4 5 6 7 8 9 10 11 12
25S (007)
18S (008)
20S pre-rRNA (PE001)

Fig. S3A

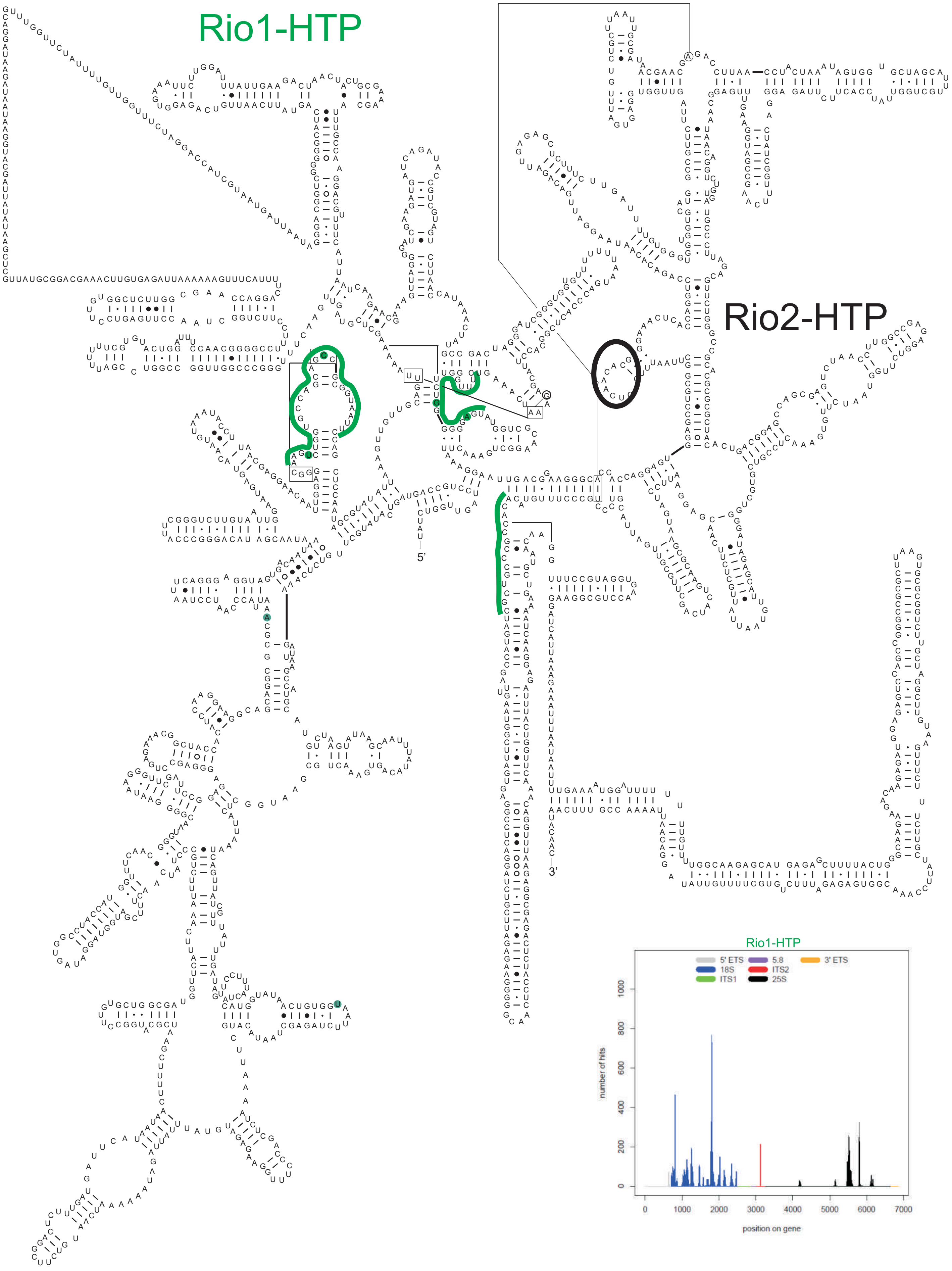

Rio1-HTP
Rio2-HTP
5'
3'
Rio1-HTP
5' ETS
5.8
3' ETS
18S
ITS2
ITS1
25S
number of hits
0
200
400
600
800
1000
position on gene
0
1000
2000
3000
4000
5000
6000
7000

Fig. S3B

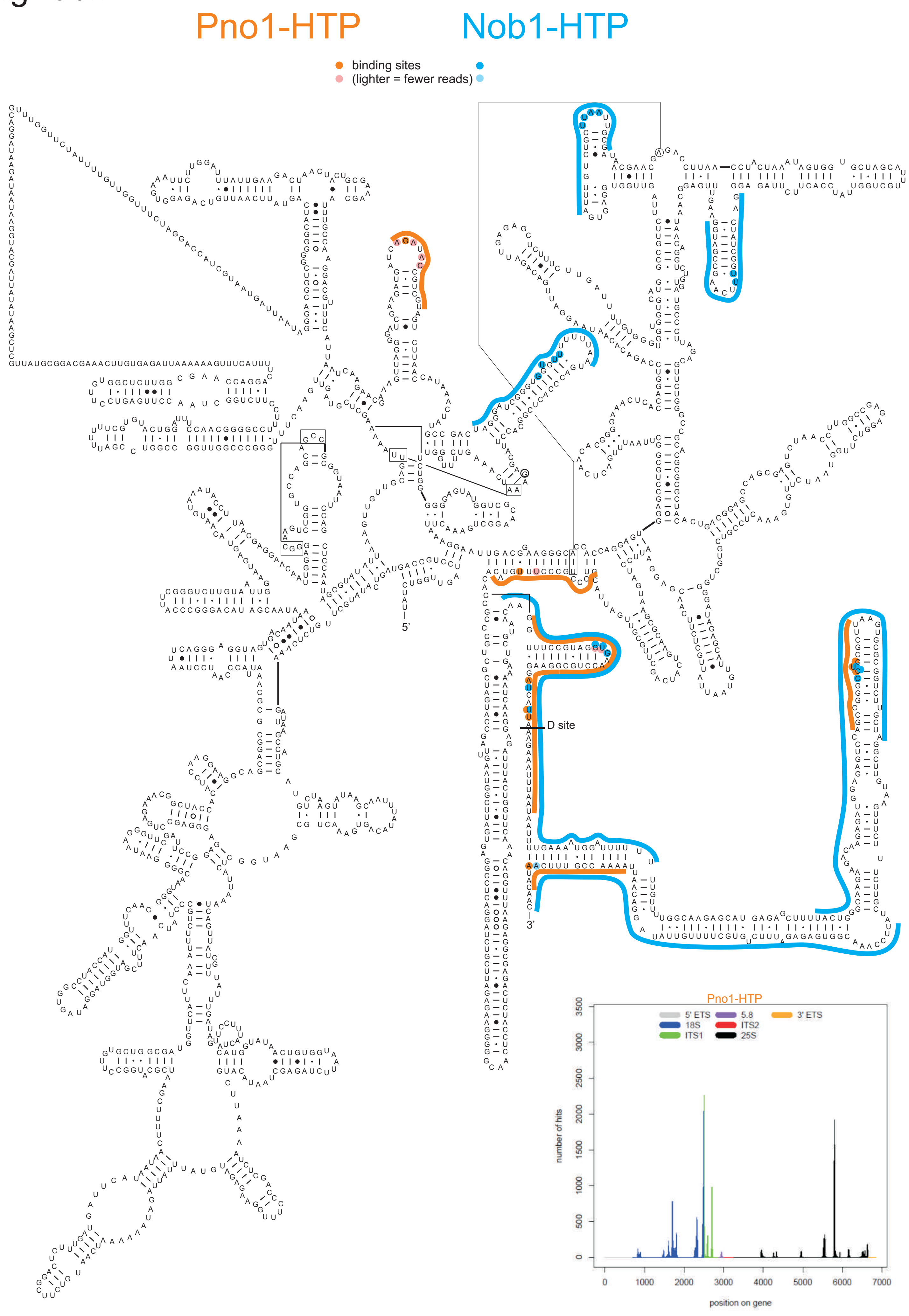

Pno1-HTP
Nob1-HTP
binding sites
(lighter = fewer reads)
D site
5'
3'
Pno1-HTP
5' ETS
18S
ITS1
5.8
ITS2
25S
3' ETS
number of hits
position on gene
0
500
1000
1500
2000
2500
3000
3500
0
1000
2000
3000
4000
5000
6000
7000
